# Histoplasmosis at a Reference Center for Infectious Diseases in Southeast Brazil: Comparison between HIV-Positive and HIV-Negative Individuals

**DOI:** 10.3390/tropicalmed8050271

**Published:** 2023-05-10

**Authors:** Ariane Gomes Paixão, Marcos Abreu Almeida, Roberta Espírito Santo Correia, Beatriz Brittes Kamiensky, Rosely Maria Zancopé-Oliveira, Márcia dos Santos Lazera, Bodo Wanke, Cristiane da Cruz Lamas

**Affiliations:** 1 Instituto Nacional de Infectologia Evandro Chagas, Fiocruz, Rio de Janeiro 21040-900, Brazil; marcos.almeida@ini.fiocruz.br (M.A.A.); rosely.zancope@ini.fiocruz.br (R.M.Z.-O.); marcia.lazera@ini.fiocruz.br (M.d.S.L.); bodo.wanke@ini.fiocruz.br (B.W.); 2 Instituto Nacional de Cardiologia, Rio de Janeiro 21040-900, Brazil

**Keywords:** histoplasmosis, Brazil, epidemiology, HIV-positive, AIDS, immunosuppression, culture, serology, mortality

## Abstract

**Objectives:** Histoplasmosis is a systemic mycosis, present globally. We aimed to describe cases of histoplasmosis (Hc) and to establish a risk profile associated with Hc in HIV-infected patients (HIV+). **Methods:** This was a retrospective study of patients with a clinical laboratory diagnosis of Hc. Data were fed into REDCap, and statistical analysis was performed with R. **Results:** We included 99 records, 65 HIV+ and 34 HIV−. Average age was 39 years. Median time from onset to diagnosis was 8 weeks in HIV− and 22 weeks in HIV+. Disseminated histoplasmosis occurred in 79.4% of HIV+, vs. 36.4% of HIV− patients. Median CD4 count was 70. Co-infection with tuberculosis was present in 20% of HIV+ patients. Blood cultures were positive in 32.3% of HIV+ vs. 11.8% of HIV− (*p* = 0.025) patients; bone marrow culture was positive in 36.9% vs. 8.8% (*p* = 0.003). Most HIV+ patients (71.4%) were hospitalized. On univariate analysis, anemia, leukopenia, intensive care, use of vasopressors and mechanical ventilation were associated with death in HIV+ patients. **Conclusions:** Most of our patients with histoplasmosis were HIV+, presenting advanced AIDS. Diagnosis was late in HIV+ patients, and they frequently presented disseminated Hc, required hospitalization, and died. Early screening for Hc in HIV+ and drug-induced immunosuppressed patients is crucial.

## 1. Introduction

*Histoplasma capsulatum* is a thermo-dimorphic and geophilic fungus that has a close relationship with environmental factors, adapting favorably in tropical and subtropical regions [1]. It has a wide geographic distribution, with autochthonous cases detected in more than 60 countries [1]. However, it has a clear predominance in the Americas, East Asia, Oceania and Sub-Saharan Africa [2]. In Latin America, histoplasmosis is the most prevalent systemic endemic mycosis [3].

*H. capsulatum* infection and the clinical expression of the disease depend on a complex interaction between the pathogen and the host, and at least three conditions are observed in the pathogenesis of this mycosis: immunocompetence of the host, virulence of the infecting strain and the acquired parasite load [4].

Immunosuppression resulting from the human immunodeficiency virus (HIV) is closely related to the disseminated form of histoplasmosis, and in 1987 it was classified as an AIDS-defining opportunistic infection by the World Health Organization (WHO) [5] and the Centers for Disease Control and Prevention (CDC) [6], emphasizing it as a potentially fatal disease. In Brazil, however, it was made notifiable in only two of the states, Rio de Janeiro and Goiás, in 2021 [7].

This study intends to describe the clinical, laboratory and epidemiological characteristics of histoplasmosis cases at Instituto Nacional de Infectologia Evandro Chagas, Fiocruz, Rio de Janeiro, Brazil, from 2000 to 2018, to compare HIV-positive (HIV+) and HIV-negative (HIV−) patients and finally to outline the associated risk profile in both groups.

## 2. Materials and Methods

We carried out a descriptive, retrospective study, by collecting data on histoplasmosis from medical records at the INI Evandro Chagas/Fiocruz, from 2000 to 2018.

Epidemiological, sociodemographic, clinical and laboratory data were extracted from outpatient visits and hospitalizations of HIV+ and HIV− patients, with the completion of a case report form specifically designed for this study.

Only definitive cases of histoplasmosis based on clinical diagnosis and laboratory evidence of histoplasmosis were included, that is, positive culture for *H. capsulatum*, histopathological finding demonstrating fungal elements consistent with *H. capsulatum* or presence of H and/or M bands by immunodiffusion and/or Western blot (WB). At the time of the study, the urinary antigen (galactomannan) test was not approved for use by the Brazilian regulatory agency, ANVISA, and was, therefore, not available for diagnosis.

We excluded pregnant women, individuals under 18 years of age, suspected cases of histoplasmosis without laboratory confirmation by means of culture, histopathology, or immunodiagnostics, as well as confirmed or probable cases with insufficient information in the medical records. Data were collected through the electronic data capture tool for research REDCap (Research Electronic Data Capture), version 12.5.5. hosted at the hospital.

Statistical analysis was performed using the R program version 3.5.1. Exploratory data analysis used absolute and relative frequencies for qualitative variables and summary measures (mean, median, first and third quartiles) for quantitative variables, depending on normality of data. Comparison between qualitative variables according to HIV status was performed using Pearson’s chi-square test or Fisher’s exact test (when necessary). The comparison between the quantitative variables according to the HIV serostatus was performed by using the Mann–Whitney test or t test (when possible). The tests used a significance level of 5% as a reference (*p* < 0.05). Univariate and multivariate analyses were performed for the outcome death due to histoplasmosis with a binomial model (logit) with the control variables in the multivariate model being age, gender, time of disease progression and HIV status; variables removed by missing data were hypoxemia, albumin, hemoglobin, AST, ALT and LDH.

The project was approved by the INI Research Ethics Committee with the number CAAE: 98094718.4.0000.5262, on 10 December 2018.

## 3. Results

An active search for histoplasmosis cases was carried out at INI, in the records of the mycology and pathological anatomy laboratories, from 2000 to 2018.

**Figure 1:**
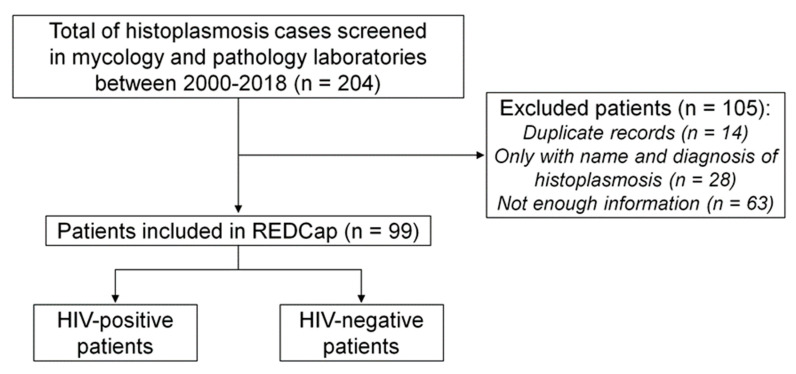
Flowchart of inclusion of patients with histoplasmosis, INI Evandro Chagas, Fiocruz, Rio de Janeiro, Brazil, 2000–2018.

In total, 204 records of patients diagnosed with histoplasmosis were found in both laboratories, but 105 were excluded (Figure 1). Of these, 14 had duplicate numbers; 28 records had only the name of the patient with a positive mycological result; 63 were records with insufficient information in the computerized system (these records basically showed just age, gender and address of the patient). Of these 63 records, 41 were men and 22 were women, with a mean age of 43.20 ± 12.17 years, 13 HIV+ and 50 HIV−. Of those excluded, 9 were empirically treated for histoplasmosis, three patients were diagnosed by radiological findings, five by histopathology, one by blood culture, one by bone marrow culture, eight by ID and 33 concomitantly by WD and ID. Ninety-nine records were then included, of which 65 were HIV+ and 34 were HIV−.

There was a predominance of white men in both groups; the mean age was 39.1 ± 11 years for HIV+ and 39.4 ± 16.1 years for HIV− patients. Just over a third of the patients had elementary education, that is, up to 8 years of schooling (HIV+ n = 26/64 or 40%; and HIV− n = 12/34 or 35.3%). Most patients in both groups came from the city of Rio de Janeiro-RJ (HIV+ n = 36/64; 55.4% and HIV− n = 18/34; 52.9%).

Use of chemotherapy drugs was reported in two HIV+ patients, one of whom had non-Hodgkin’s lymphoma, and the other had Kaposi’s sarcoma. Among HIV− patients, there were two cases of solid organ transplantation (both were using prednisone and mycophenolate) and two patients had leprosy (one of whom was using prednisone, and the other used thalidomide and had recurrent hospitalizations for pulse therapy with methylprednisolone).

Regarding the epidemiological history, HIV− patients presented, in most cases, exposure factors for the acquisition of histoplasmosis, while these were absent or infrequent in HIV+ patients. It is important to note that no findings on epidemiological history in the medical records were found in 59 (90.8%) cases of HIV+ vs. 19 (55.9%) of HIV− (*p* < 0.001). Comparing HIV+ with HIV− cases, contact with chickens was reported by one (1.5%) vs. two (5.9%) patients (*p* = 0.271); contact with bats by zero vs. six patients (17.6%, *p* = 0.001); cleaning of abandoned buildings in the last 6 months by one (1.5%) vs. two patients (5.9%), *p* = 0.271; contact with dust/soil in recent months by one (1.5%) vs. five patients (14.7%), *p* = 0.017.

Most of the HIV-positive patients with histoplasmosis in our cohort had abandoned antiretroviral therapy (18.5%) or were ART naïve (41.5%). Median CD4+ cell count was 70 cells/mm^3^ overall, and it was 42 (IQR 37,167) for those who were previously on ART vs. 76 (IQR 27,104) for those who were ART naïve, with no statistical difference between medians. Viral loads were obtained for 55/65 (84.6%) HIV-positive patients, and it was below 400 copies in 25/55 (45.4%). We did not collect information on what antiretroviral therapy they were taking, but up to 2017, the usual ART given in Brazil was tenofovir, lamivudine and efavirenz. Some patients may have been on protease inhibitors (atazanavir/ritonavir or lopinavir/ritonavir), which were usually given as alternative regimens.

Table 1 shows information on the clinical picture presented by the patients upon diagnosis. The median time between onset of symptoms and diagnosis was significantly longer for HIV+ patients, at 22 weeks, while it was 8 weeks for HIV− patients (*p* = 0.047). Most patients had fever, weight loss, and respiratory signs or symptoms. There was statistical significance regarding the frequency of hepatomegaly, splenomegaly and anemia, which were more frequent in HIV+ relative to HIV− patients.

As for the ophthalmologic evaluation, 30 HIV+ patients (46%) underwent fundoscopy, and of these, only one had a description of an eye lesion resulting from histoplasmosis infection (the report consisted of mouthpiece choroiditis, peripapillary atrophy and choroidal neovascularization). Other results showed 19 fundoscopies without alterations; four retinopathies resulting from HIV infection; two scar lesions compatible with ocular tuberculosis, three cases of cytomegalovirus retinitis, and one exam described bilateral optic nerve pallor unrelated to infection. None of the HIV− patients underwent fundoscopy.

The laboratory variables are shown in Table 2. Comparing HIV+ with HIV− patients, we observed statistical significance regarding: hypoxemia, 24.6% vs. 5.8%, (*p* = 0.041); anemia with mean hemoglobin, 9.5 (SD 2.1) vs. 12.5 (SD 2.5%), *p* < 0.01; oxaloacetic glutamic transaminase (AST), 22 (IQR 29–39) vs. 78 (IQR 39–167), *p* < 0.01; lactate dehydrogenase (Lactic Dehydrogenase, LDH), median 186 (IQR 132.8–616.2) vs. 618 (IQR 309–1304), *p* = 0.048; as well as gamma glutamyl transferase (GGT), median of 161 (IQR 81–402.5) vs. 48 (IQR 36–4894.8), *p* = 0.002. As for imaging tests, in HIV+ patients, diffuse infiltrate on plain chest X-ray predominated, and on chest CT, lymphadenopathy and nodular parenchymal lesions.

Tuberculosis was diagnosed in 13/65 (20%) HIV+ patients with histoplasmosis.

In addition to histoplasmosis, other mycological diagnoses were screened such as: cryptococcosis, aspergillosis, paracoccidioidomycosis, sporotrichosis and coccidioidomycosis. No findings were found in the HIV− group, while in the HIV+ group two immunochromatographic tests (Crag) were positive for cryptococcosis in serum, and one was positive in the cerebrospinal fluid, so cryptococcosis was diagnosed concomitantly with histoplasmosis.

Clinical specimens sent for culture are shown in Supplementary Table S1. Among the HIV+ group, specimens were collected from 39/65 (60%) patients for culture, and 28 (71.7%) were culture positive for Hc. In the HIV− group, specimens were collected from 20/34 (58.8%) patients for culture, and a positive result was verified in five patients (25%). The only two variables with statistical significance were blood culture positivity, which in HIV+ patients was 32.3% vs. 11.8% in HIV− patients (*p* = 0.025), and bone marrow culture positivity (this was 36.9% in HIV+ vs. 8.8% in HIV− patients, *p* = 0.003).

The final culture results were available for HIV+ patients in 13.5 days (IQR = 8.8–27) and for HIV− patients in 12.5 days (IQR = 7–19.5).

Table 3 shows the diagnostic tests used in the group. When comparing data from patients who were culture positive and who underwent serological tests, seven false negative immunodiagnostics were observed (non-reactive WB and non-reactive ID). All of them had the disseminated form of the disease and required hospitalization. Six of the seven patients were HIV-positive, with a median CD4 count of 39 cells/µL, all of whom had abandoned antiretroviral treatment. One of the HIV-positive patients was using chemotherapy for non-Hodgkin’s lymphoma, further worsening his immune status. The HIV-negative patient was immunosuppressed due to chronic use of corticosteroids and recent pulse therapies to control leprosy reaction. He did not receive antifungal therapy, as histoplasmosis was not suspected ante-mortem, and his positive culture became available only after he died.

As for treatment, amphotericin B deoxycholate was widely used in the HIV+ group, 32/65 (49.2%) vs. none in the HIV− group, *p* < 0.01; amphotericin B lipid complex was used in 10/65 (15.4%) of the HIV+ group vs. none of the HIV− group (*p* = 0.014). Itraconazole was the most widely used antifungal in the HIV− group.

Table 4 shows the serious outcomes and deaths of the patients with disseminated forms. More than 70% of HIV+ patients diagnosed with histoplasmosis required hospitalization; among those hospitalized, 35.5% needed to be transferred to the intensive care unit (ICU), and 40% died within approximately 6 days. In the HIV− group, only 4/34 (11.7%) were hospitalized, but 3 needed to be transferred to the ICU, 3/4 needed vasoactive drugs and mechanical ventilation, and 2/4 needed hemodialysis.

The time to finalize culture results was very close to the time between hospitalization and death among HIV− patients, and twice as long for HIV+ patients, as shown in Supplementary Table S2.

Univariate and multivariate analyses for factors associated with death in histoplasmosis are shown in Table 5. On univariate analysis, anemia, leukopenia, intensive care, use of vasopressor and mechanical ventilation were associated with death in HIV+; on multivariate analysis, only thrombocytopenia showed a near association with death.

## 4. Discussion

This study described the 19-year experience with histoplasmosis cases at INI Evandro Chagas, Fiocruz, Rio de Janeiro, a referral institute for HIV/AIDS and for systemic mycosis since the late 1980’s, with an onsite referral Mycology Reference Laboratory. Of 204 cases of histoplasmosis diagnosed in the period from 2000 to 2018, 99 were included in this study, as they presented consistent clinical and laboratory data to support analysis. Of these, approximately 2/3 were HIV+ individuals. We highlight that INI is a referral center for HIV, and this is the largest public institute, which justifies, albeit partially, the fact that the HIV+ group was almost twice as large as the HIV− group.

Sociodemographic features were similar in the HIV+ and HIV− groups, with few years of schooling and low income, which is often the case for patients utilizing the public health system in Brazil. The profile most encountered in both groups was of adult men, non-white and living in the city of Rio de Janeiro. These findings are probably justified by the majority of the INI HIV+ patients being men who have sex with men (MSM), as the HIV epidemic in Brazil affects MSM disproportionately. Brazilian statistics reported in 2021 in Unaids show that HIV seroprevalence in MSM was 18.3% [8].

An important observation in our results was the lack of reported risks of exposure to histoplasmosis in HIV+ patients; this is different from the multicenter study in Brazil, where there was exposure to bats, poultry farms and rural activities in a high proportion of patients with probable/proven histoplasmosis [9]. However, the frequency of these exposures was not different between the groups with and without histoplasmosis [9]. This leads us to suggest that a “typical” epidemiological history should not be expected or relied on to make a diagnostic hypothesis of histoplasmosis in HIV+ patients, since most of them did not report a positive epidemiological history, unlike the HIV− patients, who presented exposure to bats and dust/soil in an expressive and statistically different frequency. The absence of a relevant epidemiological history in the HIV+ group can be considered in itself as a “risk factor” for the hypothesis of histoplasmosis not being raised, resulting in a delay in diagnosis or a non-diagnosis, as we saw in the autopsy study in Manaus [10]. We noticed in our series that the diagnostic delay was longer in HIV+ patients, with a time from onset of clinical signs and symptoms to diagnosis of 22 weeks vs. 8 weeks in HIV− patients, which very likely impacts mortality.

A classical epidemiological history suggestive of Hc was absent in most HIV-positive patients in our cohort. This may have contributed to less awareness of histoplasmosis by attending physicians. Another important aspect is that the disseminated form of histoplasmosis, which is the most frequent presentation in advanced HIV, is very similar to the presentation of disseminated tuberculosis. Tuberculosis is a more familiar disease to healthcare professionals and to lay people alike, and these patients were assumed to have tuberculosis and often treated for tuberculosis, which further delayed diagnosis of histoplasmosis. The fungal burden of histoplasma is heavy in immunosuppressed patients, and their response is suboptimal, which results in the disseminated subacute form, with fever, weight loss, hepatosplenomegaly, and pancytopenia.

The disseminated clinical form in the HIV+ group was significantly more frequent than in the HIV− group (76.9% vs. 35.2%). Hepatosplenomegaly, splenomegaly, anemia, thrombocytopenia, and leukopenia were significantly more frequently found in HIV+ patients and should alert clinicians about histoplasmosis. Thrombocytopenia was not a feature of histoplasmosis in our series, differently from others [11], although it had a near association with death in our multivariate analysis.

In Brazil, in 2021, 28% and 17% of people diagnosed with HIV had a first CD4 count of less than 200 cells/mm^3^ and 100 cells/mm^3^, respectively [12]. The vast majority of HIV-infected individuals with histoplasmosis in INI were not receiving ART; a proportion had stopped HIV treatment (18.5%), and many were naïve to antiretroviral therapy (41.5%), and the median CD4+ was 70 cells/mm^3^, indicating severe immunological impairment. This finding is like other series [11,13,14]. It is well established that histoplasmosis is an opportunistic and overwhelming disease in patients with very low CD4 counts, and, therefore, we believe that screening is necessary and desirable in immunosuppressed patients, particularly those with CD4+ < 100, for both cryptococcosis and histoplasmosis, the most important systemic mycoses in HIV/AIDS. No systematic screening was performed in this retrospective series, but in patients who were ill, Western blot was requested, and, compared to the gold standard of culture results, it had a sensitivity around 90%, while double immunodiffusion had a lower sensitivity, of 65%, in HIV+ patients [15]. Although culture results, especially from blood or bone marrow in the disseminated forms, are the gold diagnostic standard, a serious problem is the prolonged time required for the completion of cultures with identification of *H. capsulatum*, which results in a risk of higher mortality for both groups. The time for the finalization of the culture results was approximately 13 days, directly impacting the outcome due to the consequent delay in the treatment with a specific antifungal. Thus, it is necessary to urgently make available other techniques that provide faster, more sensitive and specific results; in our service, we had WB as tool with internal validation [15]. In the year 2020, with the COVID-19 pandemic, our institution acquired the urinary antigen test, but this was not available to the patients studied in this report.

WB is an excellent tool that results in a highly probable diagnosis of histoplasmosis. In our study, a sensitivity of 90% in HIV+ and 93.5% in HIV− patients was observed with the WB test. Details of the cases where the WB showed a false negative result, that is, positive culture for Hc and negative WB, were sought. There were seven cases in total, six being HIV-positive and one HIV-negative. This group of patients warns us about the severity of immunosuppression in HIV-negative patients, especially in patients with leprosy reaction using high doses of corticosteroid therapy as well as HIV+ patients that may not express specific anti-histoplasma antibodies. WB for HC was later re-tested in the two surviving HIV+ patients, who by then had CD4 counts >200, and was still negative (unpublished results). It is worth noting that in clinical practice, when faced with a case with nonspecific but suggestive clinical and laboratory features of histoplasmosis, empirical antifungal treatment should be considered in the severely ill patient even if the WB result or urinary antigen test is non-reactive, while waiting for culture results. Urinary antigen testing for Hc has been shown to be more than 90% sensitive and specific in HIV-positive patients [9,14,16].

We highlight that co-infection with tuberculosis was documented in 1/5 of our HIV+ patients, which means that one should systematically investigate tuberculosis in patients with histoplasmosis, and vice versa, especially in those with severe immunodeficiency [17]. In two recent Brazilian series, concomitant TB and histoplasmosis was documented in a similar proportion of cases. In Boigues et al., 6/23 (26%) HIV-positive patients included in a hospital-based cohort in Center-West Brazil had concomitant TB-Hc [18], while in a multicenter prospective study in the five regions of Brazil, tuberculosis was diagnosed in 19/123 (15.4%) of probable/proven cases of histoplasmosis [9]. Blood cultures and bone marrow cultures are useful to diagnose disseminated tuberculosis and histoplasmosis. High sensitivity is observed when samples are processed using tests based on lysis systems (>80% sensitivity) [19]. Although histoplasmosis and TB co-infection should always be investigated, this may not always occur due to the lack of trained clinical and laboratory personnel to make definite diagnoses.

In the specific diagnosis of histoplasmosis, there was statistical significance of a higher frequency of positive blood culture (HIV+ 32.3% vs. HIV− 11.8%, *p* = 0.025) and positive bone marrow culture (HIV+ 36.9% vs. HIV− 8.8%) for *H. capsulatum*, which should encourage early bone marrow biopsy in HIV+ patients with fever, wasting syndrome and hematological changes.

Regarding treatment for histoplasmosis, amphotericin B in its deoxycholate, lipidic and liposomal forms is most often used in patients who are severely ill with the disseminated form, which is the case for immunosuppressed patients. Itraconazole is used in less severe forms, or as a consolidation treatment, following amphotericin B. Therefore, itraconazole was more often used in HIV-negative patients with less severe presentations of histoplasmosis. Outcome has more to do with the clinical presentation of the disease than with the drugs used.

We found alarming data on hospitalization rate, severity of cases in need of referral to the ICU, length of stay, and death. The hospitalization rate was more than 70% in cases of HIV and histoplasmosis, approximately a quarter of which required intensive care/vasoactive drugs/mechanical ventilation, and close to half (46%) had a fatal outcome in our series. The mortality rate for HIV–histoplasmosis coinfection was 17.7% and 13.6% in patients without HIV, according to Caceres et al. [11]. In the prospective analysis of Falci et al., overall mortality at 30 days was 22.1%, and it was lower in those who had the urinary antigen test performed (14.3%) [9]. Our retrospective study showed a disastrous scenario within a specialized service in infectious diseases prior to the availability of the urinary antigen test. Mortality in the HIV-negative group was lower, although of the few patients who were hospitalized, 3/4 (75%) died, and in one of them, histoplasmosis was not even suspected, and specific antifungal treatment was not given. On univariate analysis for risks associated with death, we found leukopenia and anemia as risk factors, which probably reflected the disseminated form of the disease in advanced immunosuppression: all deaths in our study occurred in patients with the disseminated form. Multivariate analysis did not allow for further conclusions, given the small sample size, although it did suggest that thrombocytopenia was a risk factor for death.

It is crucial to have histoplasmosis in mind when dealing with HIV-positive patients or patients who are immunodeficient due to steroid therapy or other immunosuppressive drugs, and to investigate it. This is due to the wide distribution of Hc and the environmental changes that are in place, further facilitating the spread of the microorganism. This awareness of the fungus is especially important in the Americas, in East Asia, Australasia and Sub-Saharan Africa. In our cohort, a classical epidemiological history suggestive of Hc was absent in most HIV-positive patients, a fact that highlights the importance of investigating Hc in HIV+ patients with CD4 counts below 200 cells/mm^3^ [2].

The main limitations of our study were (i) its retrospective nature, resulting in some loss of information, (ii) failure to follow up some patients at INI after diagnosis, and (iii) failure to perform necropsy in our service, which possibly resulted in not performing post-mortem diagnoses. A multivariate analysis for risk factors associated with death was not contributory due to the small sample size.

Finally, we acknowledge that a mortality rate of 46.1% in those coinfected with HIV and histoplasmosis in a service specialized in infectious disease such as INI, in a metropolis in Southeast Brazil, reveals a public health problem that is probably even greater in non-specialized services, since Brazil is an endemic country for this mycosis. The availability of the urinary antigen test will hopefully result in better performance in the near future.

## 5. Conclusions

Histoplasmosis is a neglected endemic disease, and it should be remembered especially among the group of patients who present a compatible clinical presentation and have underlying immunosuppression caused by HIV or by immunosuppressive drugs. In this subset of patients, it is an aggressive systemic mycosis, which often presents in a disseminated form and results in severe damage and death before laboratory confirmation. Therefore, there is the urgent need to build local capacity for mycology diagnosis so that patients are managed appropriately. No less importantly, we need to educate health professionals on histoplasmosis, to improve their capabilities to suspect and diagnose this severe condition. The lack of an epidemiological history compatible with histoplasmosis and the delay in diagnosing this disease, which has features similar to those of tuberculosis, are risk factors for higher mortality. The availability of composite, highly sensitive diagnostic laboratory tests, rapidity in the results and adequate treatment will favor the survival of those infected by this mycosis.

Strategies need to be implemented by the public health systems in low- and middle-income endemic countries. In Brazil, a first step in this direction has been the compulsory notification of histoplasmosis since 2021 by two of its member states, and a further step shall be extending this to all states, as well as providing public health units with the urinary antigen test for histoplasma.

## Figures and Tables

**Table 1:** Clinical findings and clinical form presented by patients diagnosed with histoplasmosis, INI 2000–2018.

	Variables		Diagnosis of HIV		
			Negative	Positive	*p*-Value
**Onset of symptoms until diagnosis: (in weeks)**		Median (IQR *)	8 (6–18)	22 (8–44)	0.047
**Signs and symptoms**	**Fever**	No	12 (35.3)	12 (18.5)	0.063
		Yes	22 (64.7)	53 (81.5)	
	**Weight loss**	No	16 (47.1)	20 (30.8)	0.11
		Yes	18 (52.9)	45 (69.2)	
	**Adenomegaly**	No	29 (85.3)	44 (67.7)	0.059
		Yes	5 (14.7)	21 (32.3)	
	**Hepatomegaly**	No	32 (94.1)	51 (78.5)	0.044
		Yes	2 (5.9)	14 (21.5)	
	**Splenomegaly**	No	33 (97.1)	53 (81.5)	0.032
		Yes	1 (2.9)	12 (18.5)	
	**Anemia**	No	30 (88.2)	42 (64.6)	0.012
		Yes	4 (11.8)	23 (35.4)	
	**Jaundice**	No	34 (100)	60 (92.3)	0.162
		Yes	0 (0)	5 (7.7)	
	**Diarrhea**	No	32 (94.1)	57 (87.7)	0.487
		Yes	2 (5.9)	8 (12.3)	
	**Vomiting**	No	33 (97.1)	54 (83.1)	0.053
		Yes	1 (2.9)	11 (16.9)	
	**Abdominal pain**	No	32 (94.1)	58 (89.2)	0.714
		Yes	2 (5.9)	7 (10.8)	
	**Respiratory syndrome**	No	15 (44.1)	33 (50.8)	0.529
		Yes	19 (55.9)	32 (49.2)	
	**Skin Lesions**	No	29 (85.3)	55 (84.6)	0.929
		Yes	5 (14.7)	10 (15.4)	
	**Oral lesions**	No	31 (91.2)	62 (95.4)	0.41
		Yes	3 (8.8)	3 (4.6)	
	**Gastrointestinal problems**	No	34 (100)	62 (95.4)	0.549
		Yes	0 (0)	3 (4.6)	
	**No symptoms reported**	No	34 (100)	61 (93.8)	0.296
		Yes	0 (0)	4 (6.2)	
**Total**			**34**	**65**	

* IQR = Interquartile Range.

**Table 2:** Results of nonspecific laboratory tests in 99 patients with histoplasmosis stratified by HIV serostatus, INI 2000–2018.

Variables		Diagnosis of HIV		
		Negative	Positive	*p*-Value
**Hypoxemia**	No	23 (67.6)	39 (60)	0.041
	No data found	9 (26.4)	9 (13.8)	
	Yes	2 (5.8)	16 (24.6)	
**Metabolic acidosis**	No	21 (63.6)	41 (65.1)	0.359
	No data found	9 (27.3)	11 (17.5)	
	Yes	3 (9.1)	11 (17.5)	
**Creatinine (mg/dL)**	Median (IQR)	0.8 (0.6–1.2)	1 (0.8–1.3)	0.091
**Urea (mg/dL)**	Median (IQR)	28.5 (17.5–31.8)	32 (26–54)	0.165
**Hemoglobin (g/dL)**	Mean (SD)	12.6 (2.5)	9.5 (2.1)	<0.001
**Total Leukocytes (cells/mm^3^)**	Median (IQR)	4800 (12.4–5810)	3410 (10.3–5647.5)	0.593
**Absolute neutrophil**	Median (IQR)	1297 (6.6–4031)	2223 (66.6–4001.2)	0.887
**Platelets (×10^3^/mm^3^)**	Median (IQR)	251 (123,877.5)	227 (117,358.5)	0.473
**ESR (mm)**	Mean (SD)	21.5 (16.3)	71.3 (36.9)	-
**CRP (mg/dL)**	Median (IQR)	8.9 (4–15.4)	9 (6.1–14.1)	0.912
**Lactate (mmol/L)**	Median (IQR)	-	1.95 (1.2–4.25)	-
**AST (IU/L)**	Median (IQR)	22 (19–39)	78 (39–167)	<0.001
**ALT (IU/L)**	Median (IQR)	34 (23–51)	41 (25–74)	0.244
**Alkaline phosphatase (IU/L)**	Median (IQR)	196.5 (99.8–219.8)	197 (150–496)	0.215
**LDH (IU/L)**	Median (IQR)	186 (132.8–616.2)	618 (309–1304)	0.048
**GGT (IU/L)**	Median (IQR)	48 (36–94.8)	161 (81–402.5)	0.002
**Albumin (g/dL)**	Median (IQR)	3.7 (2.2–4)	2.5 (2–2.9)	0.17
**INR**	Median (IQR)	-	1.345 (1.14–1.54)	-
**Total Bb (mg/dL)**	Median (IQR)	0.6 (0.4–1.1)	0.6 (0.3–1.2)	0.936
**Direct Bb**	Median (IQR)	0.1 (0.1–0.5)	0.2 (0.1–0.7)	0.066
**Indirect Bb**	Median (IQR)	0.4 (0.3–0.5)	0.4 (0.2–0.6)	0.899
**Total**		**34**	**65**	

IQR = Interquartile Range; SD = Standard Deviation; ESR = Erythrocyte Sedimentation Rate; CRP = C-Reactive Protein; IU = International Units; AST/GOT = Aspartate Aminotransferase; ALT/GPT = Alanine; Aminotransferase; LDH = Lactate Dehydrogenase; GGT = Gamma-Glutamyl Transferase; INR = International Normalized Ratio; Bb = Bilirubin.

**Table 3:** Sensitivity of diagnostic tests for histoplasmosis in HIV-positive and -negative patients, INI 2000–2018.

Variables	Diagnosis of HIV			
	Negative N = 34	Positive N = 65	Total	*p*-Value
**Cultivation**	5/20 (25%)	28/39 (71.7%)	33/59 (55.9%)	<0.01
**Histopathology**	7/12 (58.3%)	18/28 (72%)	25/40 (65.5%)	1
**Western blot**	29/32 (90.6%)	54/60 (90%)	83/92 (90.2%)	0.6095
**Double immunodiffusion**	26/32 (83.8%)	39/60 (65%)	65/94 (69.1%)	0.8924

**Table 4:** Data on hospitalization, severity, and outcome in cases of disseminated histoplasmosis, INI 2000–2018.

Variables	Diagnosis of HIV		
	Negative N = 34	Positive N = 65	*p*-Value
**Requested admission to the INI**	4 (12.1%)	45 (71.4%)	<0.001
**Referral to ICU**	3 (8.8%)	16 (24.6%)	0.104
**Use of vasoactive drugs**	3 (8.8%)	14 (21.5%)	0.189
**Hemodialysis**	2 (5.9%)	5 (7.6%)	1
**Mechanical ventilation**	3 (8.8%)	15 (23%)	0.081
**Number of deaths**	3 (8.8%)	30 (46.2%)	<0.001
**Number of deaths during hospitalization**	3 (8.8%)	18 (27.7%)	
**Hospitalization time ending in death, in days (SD)**	12 (10–14)	6 (5–18.5)	<0.001

ICU = Intensive Care Unit; SD = Standard Deviation; INI = Instituto Nacional de Infectologia Evandro Chagas (Evandro Chagas National Institute of Infectious Diseases).

**Table 5:** Univariate and multivariate analyses performed to assess factors associated with death in histoplasmosis, INI 2000–2018.

Variables		Univariate		Multivariate	
		OR * [95%IC]	*p*-Value	aOR ** [95%IC]	*p*-Value
**Age**		1.02 (0.98–1.07)	0.35	1.03 (0.97–1.1)	0.32
**Gender**	Female	1	-	1	-
	Male	0.15 (0.04–0.53)	<0.01	0.08 (0.01–0.4)	<0.01
**Disease progression time**		1 (0.97–1.02)	0.74	1 (0.96–1.04)	0.92
**HIV diagnosis**	Negative	1	-	1	-
	Positive	7.47 (0.93–60.16)	0.06	8.09 (0.87–75.55)	0.07
**Weight loss**	No	1	-	1	-
	Yes	1.33 (0.38–4.68)	0.65	1.47 (0.25–8.59)	0.67
**Splenomegaly**	No	1	-	1	-
	Yes	1.24 (0.24–6.35)	0.8	1.67 (0.19–14.87)	0.65
**Anemia**	No	1	-	1	-
	Yes	5.64 (1.65–19.26)	**0.01**	4.03 (0.81–20.08)	0.09
**Leukopenia**	No	1	-	1	-
	Yes	6.51 (1.82–23.3)	**<0.01**	3.65 (0.7–19.11)	0.12
**Thrombocytopenia**	No	1	-	1	-
	Yes	2.57 (0.59–11.09)	0.21	6.92 (0.97–49.56)	0.05
**ICU admission**	No	1	-	1	-
	Yes	19.25 (3.52–105.29)	**<0.01**	-	-
**Use of vasoactive catecholamines**	No	1	-	1	-
	Yes	73.33 (13.13–409.52)	**<0.01**	-	-
**Hemodialysis**	No	1	-	1	-
	Yes	2.95 (0.51–17.06)	0.23	5.44 (0.43–68.86)	0.19
**Mechanical ventilation**	No	1	-	1	-
	Yes	62.07 (11.42–337.52)	**<0.01**	-	-

* OR = Odds Ratio; ** aOR = Adjusted Odds Ratio. Binomial model (logit); Outcome: death due to histoplasmosis. Control variables in the multivariate model: age, sex, disease progression time and HIV diagnosis. Variables removed by missing values: clinical form, hypoxemia, albumin, hemoglobin, AST, ALT, LDH.

## Data Availability

Data has not been made publicly available due to ethical reasons.

## Supplementary Materials

The following supporting information can be downloaded at: https://www.mdpi.com/article/10.3390/tropicalmed8050271/s1, Table S1: Clinical specimens collected for culture in patients diagnosed with histoplasmosis, INI 2000–2018.; Table S2: Temporal comparison between obtaining the result of the culture and the date of hospitalization until death, INI 2000–2018.
