# Supplementary material for: Histoplasmosis at a Reference Center for Infectious Diseases in Southeast Brazil: Comparison between HIV-Positive and HIV-Negative Individuals
Source: Trop Med Infect Dis. 2023 May 10;8(5):271. doi: 10.3390/tropicalmed8050271 (PMC10220818; doi:10.3390/tropicalmed8050271)
Supplement: Supplementary file 1 [file tropicalmed-08-00271-s001.zip › tropicalmed-2380650-supplementary.pdf]

Supplementary

Table S1. Clinical specimens collected for culture in patients diagnosed with histoplasmosis, INI 2000–2018.

	Variables		Diagnosis of HIV		
			Negative	Positive	*p*-value
Culture	Lymphnode biopsy	No	34 (100)	58 (89.2)	0.092
		Yes	0 (0)	7 (10.8)	
	Blood	No	30 (88.2)	44 (67.7)	0.025
		Yes	4 (11.8)	21 (32.3)	
	Bone Marrow	No	31 (91.2)	41 (63.1)	0.003
		Yes	3 (8.8)	24 (36.9)	
	Skin Biopsy	No	31 (91.2)	61 (93.8)	0.689
		Yes	3 (8.8)	4 (6.2)	
	Bronchoalveolar Lavage	No	29 (85.3)	61 (93.8)	0.268
		Yes	5 (14.7)	4 (6.2)	
	Sputum	No	31 (91.2)	60 (92.3)	1
		Yes	3 (8.8)	5 (7.7)	
	Urine	No	34 (100)	64 (98.5)	1
		Yes	0 (0)	1 (1.5)	
	Negative culture	No	27 (79.4)	54 (83.1)	0.653
		Yes	7 (20.6)	11 (16.9)	
	**Total**		**34**	**65**	

Table S2. Temporal comparison between obtaining the result of the culture and the date of hospitalization until death, INI 2000–2018.

Variables		Diagnosis of HIV		
		Negative	Positive	*p*-value
**Time to receive culture result (in days)**	Median (IQR)	12.5 (7-19.5)	13.5 (8.8-27)	0.292
**Hospitalization time resulting in death**	Mean (SD)	12 (10,14)	6 (5,18.5)	< 0.001

IQR = Interquartile Range; SD = Standard Deviation
